# Self-Assembled Thermoresponsive Nanogel from Grafted Hyaluronic Acid as a Biocompatible Delivery Platform for Curcumin with Enhanced Drug Loading and Biological Activities

**DOI:** 10.3390/polym13020194

**Published:** 2021-01-07

**Authors:** Jittima Amie Luckanagul, Pahweenvaj Ratnatilaka Na Bhuket, Chawanphat Muangnoi, Pranee Rojsitthisak, Qian Wang, Pornchai Rojsitthisak

**Affiliations:** 1 Department of Pharmaceutics and Industrial Pharmacy, Faculty of Pharmaceutical Sciences, Chulalongkorn University, Bangkok 10330, Thailand; jittima.luck@gmail.com; 2 Natural Products for Ageing and Chronic Diseases Research Unit, Chulalongkorn University, Bangkok 10330, Thailand; bpahweenvaj@gmail.com (P.R.N.B.); pranee.l@chula.ac.th (P.R.); 3 Cell and Animal Model Unit, Institute of Nutrition, Mahidol University, Nakhon Pathom 73170, Thailand; chawanphat.mua@gmail.com; 4 Metallurgy and Materials Science Research Institute, Chulalongkorn University, Bangkok 10330, Thailand; 5 Department of Chemistry and Biochemistry, University of South Carolina, 631 Sumter St., Columbia, SC 29208, USA; WANG263@mailbox.sc.edu; 6 Department of Food and Pharmaceutical Chemistry, Faculty of Pharmaceutical Sciences, Chulalongkorn University, Bangkok 10330, Thailand

**Keywords:** curcumin, hyaluronic acid, HA-pNIPAM, thermoresponsive nanogel

## Abstract

A hyaluronic acid-grafted poly(*N*-isopropylacrylamide) (HA-pNIPAM) was synthesized as a polymeric nanogel platform for encapsulation and delivery of hydrophobic bioactive compounds using curcumin as a model drug. As demonstrated by transmission electron microscopy and dynamic light scattering techniques, the HA-pNIPAM was simply assembled into spherical nano-sized particles with the thermoresponsive behavior. The success of curcumin aqueous solubilization was confirmed by fluorescent spectroscopy. The resulting nanogel formulation enhanced the aqueous solubility and uptake into NIH-3T3 cells of curcumin. This nanogel formulation also demonstrates cytocompatibility against NIH-3T3 cells, which deems it safe as a delivery vehicle. Moreover, the formulation has a slight skin-protection effect using an artificial skin equivalence model. The curcumin-loaded HA-pNIPAM nanogel showed an anti-proliferative activity against MDA-MB-231, Caco-2, HepG2, HT-29, and TNF-α-induced hyperproliferation of keratinocyte (HaCaT) cells. The thermoresponsive HA-pNIPAM nanogel reported here could be further optimized as a platform for controlled-release systems to encapsulate pharmaceuticals for therapeutic applications.

## 1. Introduction

Hyaluronic acid (HA) is a natural polymer abundant in nature as a ubiquitous component in the connective tissue microenvironment for cell survival, proliferation, motility, and differentiation [1,2]. In the human body, the synovial fluid and chondrocytes’ extra-cellular matrix are mainly composed of HA that functions as a lubricant and structural support [3]. Functionalization of HA can be achieved for various applications with different methods and approaches. Some HA derivatives are well-established and commercialized. With the current progress in research and development, native unmodified HA is being replaced in biomedical applications by chemically modified HA with its specific characteristics for drug delivery or tissue engineering platforms [4]. A carboxyl group on D-glucuronic acid allows HA functionalization while its biological properties remain. The amide bond formation is especially feasible for the HA conjugation and polymer backbone grafting. The amide formation can be achieved by activating carboxyl groups of HA with *N*-(3-dimethylaminopropyl)-*N*’-ethylcarbodiimide hydrochloride (EDC) and *N*-hydroxysuccinimide (NHS). The reaction involves the formation of an O-acylisourea upon the activation with EDC forming the stabilized *N*-acylurea [5].

The use of phytochemicals as therapeutic agents is highly considered in pharmaceutical research and is extensively explored in chemotherapeutic applications [6,7]. Curcumin, a polyphenol derived from the rhizome of *Curcuma longa* L., is one of the promising naturally derived compounds because of its wide range of biological uses [8]. Curcumin has been investigated for its usage in chemopreventive and chemotherapeutic applications in several cancers such as colon, breast, and liver cancers [9,10]. Curcumin also has shown anti-psoriatic activity in mouse models [11,12]. However, curcumin has poor oral bioavailability due to its chemical instability, low aqueous solubility, and rapid metabolism, all of which hinder it from being developed as a therapeutic agent [13]. A high dose of curcumin is, therefore, required to achieve its desirable pharmacological effects. For example, curcumin at a dose of 1000 mg/day showed an improvement in Disease Activity Score (DAS) and American College of Rheumatology (ACR) scores in patients with active rheumatoid arthritis [14].

To overcome the limitations of curcumin, several nanotechnology-based drug carriers have been developed to encapsulate and deliver curcumin, including liposomes, polymeric nanoparticles, polymeric micelles, solid lipid nanoparticles, macromolecular conjugates, and hydrogels [15]. Moreover, the responsive biocompatible delivery system has been widely explored for effective therapeutic applications to improve drug bioavailability. Rao et al. reported that hybrid halloysite nanotubes as multifunctional biocompatible nanoparticle responsive delivery system of curcumin for anti-cancer activity [16]. These nanoparticulate systems can improve pharmacokinetics, physicochemical properties, and tissue targeting, allowing curcumin to exert desirable pharmacological effects at the site of action. For instance, a curcumin-loaded solid lipid nanoparticle formulation was orally administered to rats with cerebral ischemia conditions at doses of 25 mg/kg/day and 50 mg/kg/day for eight days [17]. This nanoformulation improved the cognition and neurological scoring in the rats under study, increased the endogenous antioxidant enzyme levels, and inhibited the lipid oxidation process. Additionally, the curcumin-loaded solid lipid nanoparticles prolonged the circulation time of curcumin and increased curcumin accumulation in the brain by 30 times compared to the unformulated curcumin. This evidence demonstrates the potential of nanotechnology to deliver curcumin with improved bioavailability and targeting.

Nanogels are nanometric-sized, three-dimensional polymeric networks that possess the properties of hydrogels and nanoparticles. They have high water content, tunable structures, and biocompatibility properties. Nanogels formed from hydrophilic polymers can offer encapsulation platforms to deliver pharmaceuticals, particularly hydrophobic compounds and biomacromolecules [18]. Naturally derived polysaccharides, such as chitosan and hyaluronic acid, have been used to form nanogels for biomedical applications because of their hydrophilicity, biocompatibility, functionality, and biodegradation properties [19,20,21]. They can be functionalized or fine-tuned to provide a stimuli-responsive function by conjugating stimuli-responsive moieties to their functional groups, e.g., –OH, –NH_2,_ and –COOH. Poly(*N*-isopropylacrylamide) or pNIPAM is a hydrophilic polymer with a thermosensitive property. It has a low critical solution temperature (LCST) of 32 °C. The sol-gel transition at this temperature is useful for biomedical applications since it is between room and body temperatures, which can be adjusted for tissue engineering or controlled-release drug delivery purposes by incorporation of comonomers or hydrophilic groups [22]. Recently, we successfully prepared thermo-responsive nanogels from pNIPAM-grafted chitosan as a vehicle for delivery of curcumin to the cancer cells [23]. The grafting process was achieved via the amide bond formation between the –NH_2_ group of chitosan and –COOH yielding pNIPAM-grafted chitosan nanogels with the sharp phase transition close to 37 °C.

**Figure 1:**
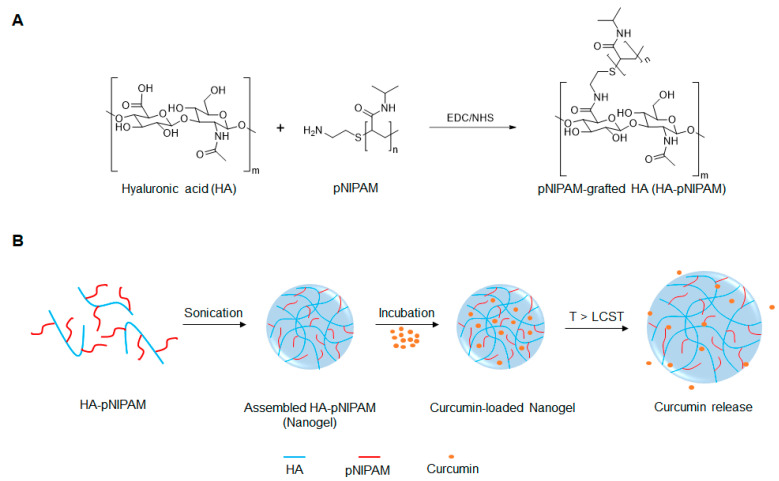
Schematic representation of (**A**) the preparation of pNIPAM-grafted hyaluronic acid (HA-pNIPAM) and (**B**) the assembly of HA-g- pN nanogel and curcumin release.

To the best of our knowledge, few reports exist on the development of thermo-responsive HA-pNIPAM polymer for drug delivery [24,25,26]. We initially reported on the HA modified with pNIPAM nanogel, pNIPAM-grafted hyaluronic acid (HA-pNIPAM), for curcumin formulations and showed the behavior of nanogels in different solvent systems, the method for curcumin loading, and curcumin stabilization via the nanogel system [27]. In the present study, we further investigated the thermoresponsive HA-pNIPAM nanogel for curcumin delivery, using HA as a backbone grafting with a low molecular weight pNIPAM at a 5% degree of molar substitution (DS). The schematic design for the development of the HA-pNIPAM nanogel system is depicted in Figure 1. The safety and efficacy of the prepared HA-pNIPAM nanogels containing different amounts of curcumin were also evaluated.

## 2. Materials and Methods

All chemicals were purchased from commercial suppliers and used as- is unless otherwise noted. Curcumin was synthesized using the previously published method, and the resulting product was structurally confirmed by ^1^H NMR [28].

### 2.1. Synthesis of Drug-Free HA-pNIPAM Nanogel

The EDC/NHS coupling reaction for the amide conjugation was used to synthesize HA-pNIPAM. The synthesis protocol was described in our previous report [27] with some modifications. Briefly, sodium HA (M.W. 47 kDa) at 1% *w/v* in ultra-pure water was prepared, followed by the addition of pNIPAM (M.W. 5500 Da). The molar ratio of HA subunit:pNIPAM was 1:0.05. A four-fold molar excess of EDC and NHS was added. During the first hour, the reaction was maintained for the specific pH at 5.5 ± 0.3. Then, the pH was adjusted to 7.5 ± 0.3 using NaOH solution. The reaction was run at room temperature for different time periods for optimization. After the reaction was stopped, the resulting products were purified via dialysis for three days and further freeze-dried. The purified products were subjected to structural analysis by ^1^H NMR using D_2_O as a solvent. ^1^H NMR spectra were recorded using a JEOL JNM-A500 500 MHz spectrometer (JEOL Tokyo, Japan) at 70 °C. The grafting ratio of the synthesized product was calculated from its ^1^H NMR spectra. Thermogravimetric analysis (TGA) was carried out using a TGA Q5000 V3.15 Build 263, TA Instrument, New Castle, DE, USA

### 2.2. Preparation of Curcumin-Loaded HA-pNIPAM Nanogel

The curcumin-loaded HA-pNIPAM nanogel was prepared according to our previous report [27] with some modification. In this study, the buffer system was used as a solvent instead of pure water. The HA-pNIPAM nanogel was prepared at 0.5% *w*/*v* in phosphate-buffered saline (PBS) following sonication for 15 min, settling nanogels overnight at 4 °C, and centrifugation at 3000× *g* for 5 min. The resulting nanogel was designated as HA-pNIPAM 05. Loading of curcumin into the nanogel was achieved by a simple incubation method. In brief, 100 µM curcumin in ethanol was added dropwise into the HA-pNIPAM 05 solution under a constant stir at 500 rpm. The vessel for curcumin loading was kept at 4 °C protected from light. After incubation for 24–48 h, the unloaded curcumin was removed by centrifugation at 3000× *g* for 5 min. The resulting solution was then centrifuged to remove free curcumin. The resulting curcumin-loaded nanogel formulation was termed CUR-HA-pNIPAM 05.

### 2.3. Characterization of Drug-Free and Curcumin Nanogel

The amounts of curcumin were measured by fluorescent spectroscopy. The amount of curcumin loading was determined and calculated against a calibration curve, and the loading capacity of the formulation was calculated as follows:

Loading Capacity (%) = 100 (Mole_drug_/Mole_polymer_)

The loading capacity demonstrates the capability of the polymer to hold curcumin as the molar ratio based on the polymer’s dry weight.

The particle size of the HA-pNIPAM 05 nanogel was determined by the dynamic light scattering (DLS) technique. To examine the thermoresponsive behavior of the nanogel, average sizes of nanogel particles were measured using a controlled temperature program ranging from 4–40 °C with an increasing rate of 1 °C/min. The abrupt change in temperature at the size of the nanogel particles was considered as the LCST of the nanogel. The morphology of the nanogel was evaluated by transmission electron microscopy (TEM) using a JEOL JEM-2100 (JEOL, Tokyo, Japan).

### 2.4. Confocal Cell Uptake Studies

Acid-etched coverslips placed in 24-well plates were seeded with NIH-3T3 cells at a seeding density of 20,000 cells per coverslip. The 24-well plates were incubated for 24 h, allowing the cells to attach to the well. After 24 h incubation, the Dulbecco’s Modified Eagle Medium (DMEM) containing 10% *v*/*v* fetal bovine serum (FBS) and 1% *v*/*v* penicillin-streptomycin was removed, and the cells were washed with a PBS buffer solution. Then, nanogel particles in the culture medium were added to each well at the final drug concentration of 3 µM in triplicate, and the treated cells were further incubated for 6 h. After incubation for 6 h, the media with the nanogel sample were removed, and the coverslips were washed with a PBS buffer solution. The cells were fixed with 3.7% paraformaldehyde, followed by a final PBS wash. The coverslips were air-dried and mounted onto glass slides using DPX mounting reagent (Sigma-Aldrich, St. Louis, MO, USA). Subsequently, the cells were stained with 4′,6-diamidino-2-phenylindole (DAPI) and rhodamine-phalloidin to visualize the nucleus and actin. The samples were then observed under a confocal microscope (Olympus DSU-IX81 Spinning Disc Confocal, Olympus, Tokyo, Japan) to investigate the cellular internalization of CUR-HA-pNIPAM 05.

### 2.5. Cell Viability Assay

The cell viability of NIH-3T3 cells was assessed using a CellTiter-Blue^®^ (CTB) cell viability assay (Promega, San Luis Obispo, CA, USA). After treating NIH-3T3 cells with curcumin, HA-pNIPAM 05 nanogel, and CUR-HA-pNIPAM 05 nanogel, the media containing the samples in each well (in a 96-well plate format) were replaced with a 100 μL of the pre-heated medium containing 10% CTB and incubated at 37 °C for 1 h under 5% *v*/*v* CO_2_. The medium containing 10% CTB was used as a negative control. The fluorescence intensity of a CTB metabolite was measured at the excitation wavelength of 560 nm and the emission wavelength of 590 nm using a microplate reader (SpectraMax^®^ M2, Molecular Devices, San Jose, CA, USA).

### 2.6. Cytotoxicity of Curcumin Nanogel Formulation

Cytotoxicity of CUR-HA-pNIPAM 05 was investigated against MDA-MB-231, Caco-2, HepG2, and HT-29 cells. All cell lines used in this study were obtained from the American Type Culture Collection (ATCC, Rockville, MD, USA). Cells were seeded to a 96-well plate at a density of 10,000 cells/cm^2^ and incubated at 37 °C in a humidified atmosphere with 5% *v*/*v* CO_2_. The cells were cultured in different complete media. The complete medium for Caco-2 cells comprised of DMEM, 10% *v*/*v* heat-inactivated FBS, 1% *v*/*v* penicillin-streptomycin, 1% *v*/*v* L-glutamine, 1% *v*/*v* non-essential amino acid, and 0.2% *v*/*v* fungizone. MDA-MB-231 and HT-29 cells were cultured in DMEM containing 10% *v*/*v* heat-inactivated FBS and 1% *v*/*v* penicillin-streptomycin. For HepG2 cells, the complete medium consisted of 10% *v*/*v* FBS, 1% *v*/*v* penicillin-streptomycin, and DMEM. The CUR-HA-pNIPAM 05 nanogel was prepared at four concentrations using the culture medium as a diluent. The unloaded HA-pNIPAM 05 and a curcumin solution were also prepared at the same dilutions. Cells in the corresponding culture medium were used as negative controls, while cells treated with doxorubicin were used as positive controls. The cytotoxicity of the samples was assessed using the 3-(4,5-dimethylthiazol-2-yl)-2,5-diphenyl-2H-tetrazolium bromide (MTT) assay.

### 2.7. In Vitro Acute Skin Irritation Assay

The reconstructed human epidermis (RHE) model was used to evaluate the skin irritation after applying the CUR-HA-pNIPAM 05 nanogel formulation. SkinEthic™-RHE, batch number 17/RHE 064, was purchased from EPISKIN Laboratories, Lyon, France. SkinEthic™-RHE was listed as one of the validated alternative methods compliant to the OECD Test Guideline 431 (meeting performance standards 2006) and Commission Regulation (EC) No 440/2008. The skin model was acknowledged as human skin equivalents (HSEs) that can be applied as an evaluation platform in the skin corrosivity and skin irritation tests. SkinEthic™-RHE was pre-incubated with the growth culture medium provided by EPISKIN Laboratories for 24 h at 37 °C in the 12-well tissue culture plate. After 24 h pre-incubation, the models were transferred to the new medium. The nylon mesh was placed onto the skin model, after which 30 µL of the sample was applied to cover the epidermis surface of each model uniformly. The models were then post-incubated on the growth culture medium for 24–42 h at 37 °C. At the end of the treatment incubation, the models were rinsed with a PBS solution. To preserve the tissue model for further histological analysis, the tissue was removed from the trans-well plastic insert and transferred to the tube prefilled with 4% paraformaldehyde. The fixed tissue was preserved in 4% paraformaldehyde at 4 °C before embedded in paraffin. Sections of 3 μm were stained with hematoxylin and eosin (H&E) for general histological evaluations.

### 2.8. Evaluation of the Anti-Psoriatic Effect

HaCaT cells were seeded at a density of 5 *×* 10^3^ cells in a 96-well plate and incubated at 37 °C in a humidified atmosphere enriched with 5% *v*/*v* CO_2_. After seeding for 24 h, cells were washed with serum-free medium. TNF-α in serum-free medium was added to each well except the control group at a final concentration of 10 ng/mL. The TNF-α-treated cells were incubated for 24 h. The cultured cells were washed and added with a serum-free medium. Subsequently, the cells were treated with curcumin or CUR-HA-pNIPAM 05 nanogel formulation at the final curcumin concentration of 1 and 5 µg/mL. The serum-free medium containing 0.5% of DMSO and the nanogel without curcumin was used as the control group. After incubation for 24 h, the treated cells were washed with serum-free medium and were added with an MTT solution in PBS at 0.5 mg/mL. The incubation was continued for another 4 h. The medium was removed, and 200 µL of DMSO was then added to each well to dissolve the formazan crystals. The absorbance of formazan was measured at 540 nm by a microplate reader (SPECTROstar, BMG LABTECH GmbH, Ortenberg, Germany). The experiment was performed in four replicates. The results are presented as % cell viability in comparison with the control.

### 2.9. Statistical Analysis

All experiments were performed in triplicate, and the results are expressed as mean ± standard deviation. Statistical analysis was performed by *t*-test for comparing the loading capacity while one-way ANOVA was used in cell experiments. The *p* ≤ 0.05 was used to indicate a significant difference in the statistical results.

## 3. Results and Discussion

### 3.1. Preparation of Control and Curcumin Loaded Nanogels

The preparation of CUR-HA-pNIPAM involved 3 steps; (1) optimized coupling reaction of pNIPAM onto hyaluronic acid, (2) nanogel particle formation by sonication, and (3) introducing curcumin further purification by centrifugation. Steps 2 and 3 were performed following the previously reported study [27]. The additional optimization of the synthesis process was completed in this study. In polymer grafting, the structure of each grafting reaction product was analyzed using ^1^H NMR. As shown in Figure S1 in the supplementary material, a 5% degree of modification was carried out from various running times from 24 h up to five days. A 0.5% *w*/*v* of grafted HA-pNIPAM polymer was then used to form a nanogel formulation designated as HA-pNIPAM 05. The formulation was then incubated at 4 °C with excess curcumin overnight before further characterizations.

### 3.2. Thermal Analysis

**Figure 2:**
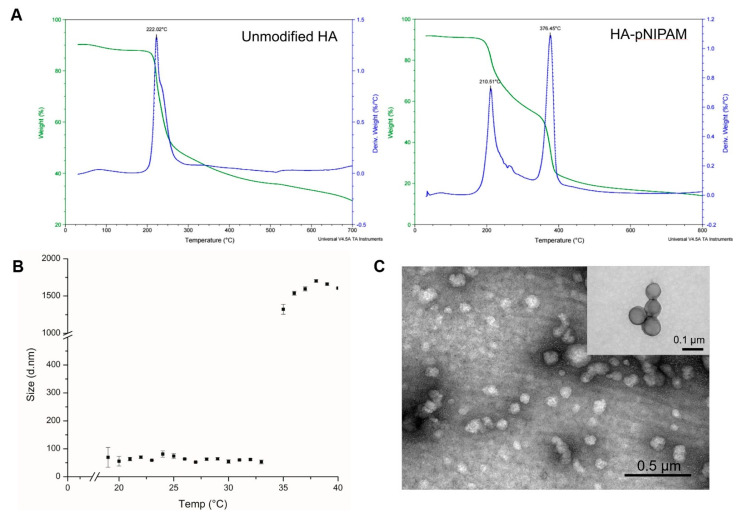
The characterization of HA-pNIPAM nanogel. (**A**) the thermal degradation profile of pNIPAM-grafted HA, in which the green line shows the total weight reduction; the blue line shows the derived weight reduction profile representing the grafted-polymer that decomposed at two different temperatures. (**B**) Particle size measured by DLS shows the thermoresponsive behavior of the HA-pNIPAM 05 nanogel. (**C**) The corresponding TEM micrograph represents the spherical morphology of the nanogel at 25 °C (negatively stained) with an inset image from the higher magnification TEM (positively stained). The average size of the particle strikingly changed at the LCST.

The thermal decomposition of the prepared systems was investigated by TGA, which is given in Figure 2A. It shows that the initial degradation temperature of the controlled HA nanogel was very close to 200 °C. The degradation profile of the grafted HA was different from the parent unmodified HA. Two layers of degradation temperatures appeared, which demonstrates that the polymer possessed two major components with different degradation temperatures. Thus, the TGA curve of the grafted HA polymer could represent the successful grafting of pNIPAM.

Figure 2B,C show the HA-pNIPAM 05 nanogel with the stable dispersion in the water at a polymer concentration at 0.5% *w*/*v* in a PBS solution. The TEM images of Figure 2C and its inset were taken from different grids with different samples at each time of analysis. Since this particulate system is formed as a swellable polymer matrix in phosphate buffer, it is highly likely that the phosphate salt could interfere with the staining process [29]. The thermoresponsive behavior of the formulation was present. At below 19 °C, the size of the nanogel was unstable. The particles size below 100 nm were observed between 19–33 °C. Then, the nanogel particles were disintegrated when the temperature increased above 33 °C.

### 3.3. Size, Morphology, and Drug Loading of the Nanogel Formulation

**Figure 3:**
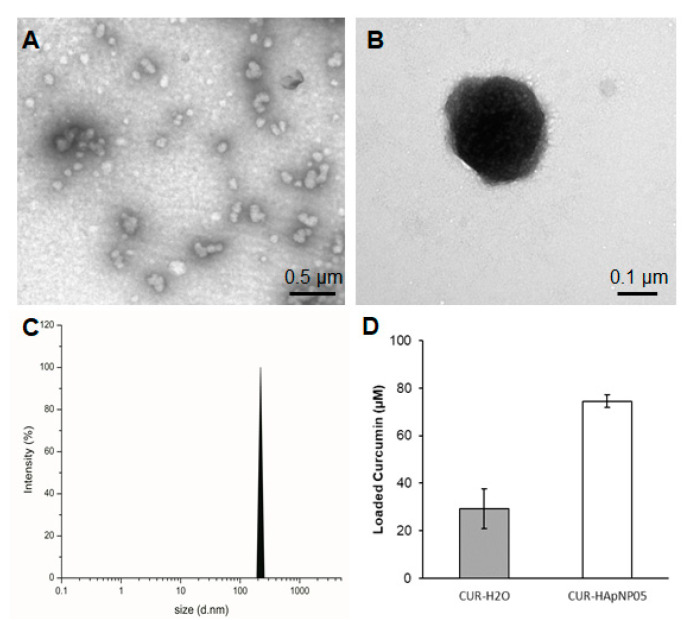
Size, morphology, and loading capacity of CUR-HA-pNIPAM 05 formulation. (**A**) TEM micrograph of CUR-HA-pNIPAM 05 formulation shows that the particles were sized around 100–300 nm. (**B**) A magnified TEM image for a single CUR-HA-pNIPAM 05 nanogel particle; (**C**) DLS results demonstrate the curcumin nanogels in the size range correlated with TEM at PDI = 0.2. (**D**) Loading capacity of the formulation compared to the curcumin in aqueous solution. Scale bars indicate 0.5 µm for (**A**) and for 0.1 µm for (**B**). The values expressed in (**D**) are means ± SD, n = 3.

Sizes of the nanostructure with loaded curcumin were evaluated based on the TEM image and DLS spectra of HA-pNIPAM 05 and are shown in Figure 3. TEM analysis confirmed that the CUR-HA-pNIPAM 05 nanogel was spherical in shape with a size that ranged around 100–300 nm (Figure 3A,B). DLS data also supported the size of CUR-HA-pNIPAM 05 nanogel observed by TEM with the polydispersity index (PDI) of 0.2 (Figure 3C). The narrow size distribution was observed with the incorporation of curcumin in this system. Although it is unusual for self-assembled nanoparticulate systems, the narrow size distribution after incorporating payloads or different components onto the nanogel backbone has been reported by several researchers. For example, Iwasaki et al. showed their self-assembled nanogel system, in which the average size of nanogel particles was 124 ± 2 nm, and the nanogels were mono-dispersed with the PDI of 0.21 [30]. Another recent report by Rusu et al. claimed that the assembly behavior of nanogels based on maleic anhydride chitosan derivatives and bovine serum albumin relied on the presence of hydrophobic segments on the protein macromolecular chain, which stabilized the nanogels. One assembly condition of their nanogel system yielded a hydrodynamic diameter of 210 nm with a PDI of 0.169 [31].

Figure 3D shows the enhanced loading capacity of curcumin in the HA-pNIPAM 05 nanogel experimented at 0.5% *w*/*v* polymer concentration compared to the formulation without the polymer. The loading capacity of the grafted HA was calculated more than 850 times of curcumin molecule per its polymeric chain in the nanogel structure. Statistical analyses were performed to compare the loading capacity between the HA-pNIPAM 05 nanogel and the control. The loading capacity of the CUR-HA-pNIPAM 05 was significantly higher than the control (*p* < 0.05). The curcumin concentration in the HA-pNIPAM 05 nanogel was found at 75 µM, while the curcumin concentration in PBS (control) was 30 µM. The solubility improvement is consistent with other nanogel systems. For example, Gonçalves et al. reported the curcumin solubility in an aqueous-based nanogel composed of a hydrophilic dextrin backbone with grafted acrylate groups that could help curcumin solubility up to 50 μM with 0.2% nanogel. The loading efficiency was also concentration-dependent [32]. Bang et al. also reported that the solution of 1% nanogels of acetylated ulvan could increase curcumin solubility to 641 μM [33]. Therefore, the developed nanogel system could slightly increase the aqueous solubility of curcumin and had the potential for encapsulating other hydrophobic molecules. Further formulation optimization should be performed in future studies, which would extend the applications in pharmaceutical aspects.

### 3.4. Safety and Cellular Uptake of CUR-HA-pNIPAM 05

**Figure 4:**
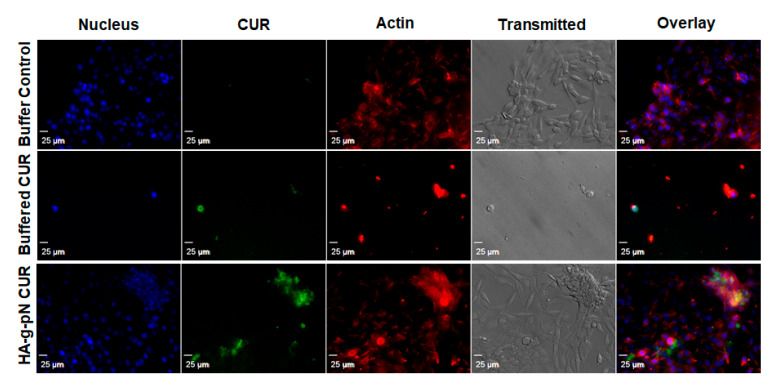
Confocal microscopy shows the cellular drug uptake in NIH-3T3 cells. DAPI (nuclear staining) and rhodamine-phalloidin (actin staining) were used as counter-stains. The images were obtained using Olympus IX81 with DSU confocal mode under a 20× oil lens (NA = 0.85). Exposure times were kept constant for all samples (500 ms for DAPI and rhodamine-phalloidin, 1000 ms for curcumin).

The toxicity of nanogel formulations was evaluated via CTB cell viability assay on NIH 3T3. Figure S2 in the supplementary material demonstrates the safety of the polymeric material itself as a vehicle for drug delivery when tested with the formulation containing 0.5% *w*/*v* of the HA-pNIPAM. No significant changes in cell viability were observed across the treatment dilutions except the highest dilution containing 75 µM and 30 µM curcumin in the HA-pNIPAM 05 nanogel and the solution, respectively. This result is interesting since a two-times higher dose of curcumin loaded in the nanogel resulted in the same cell viability level as in the solution form. This observation could attest to improved safety as a benefit for the curcumin nanogel formulation. Figure 4 illustrates the cellular uptake and localization of curcumin in the cells cultured as a monolayer. Curcumin (green fluorescence) was shown to be uptaken by fibroblast (NIH-3T3), and the localization was confirmed with the counter-stains for cell structures with DAPI (nuclear staining) and rhodamine-phalloidin (actin staining). The confocal imaging experiment showed that the fabricated HA-pNIPAM 05 could reduce the drug toxicity toward NIH-3T3 while the cellular uptake of curcumin was allowed, as demonstrated by the localization of the green fluorescence of curcumin within the cells.

**Figure 5:**
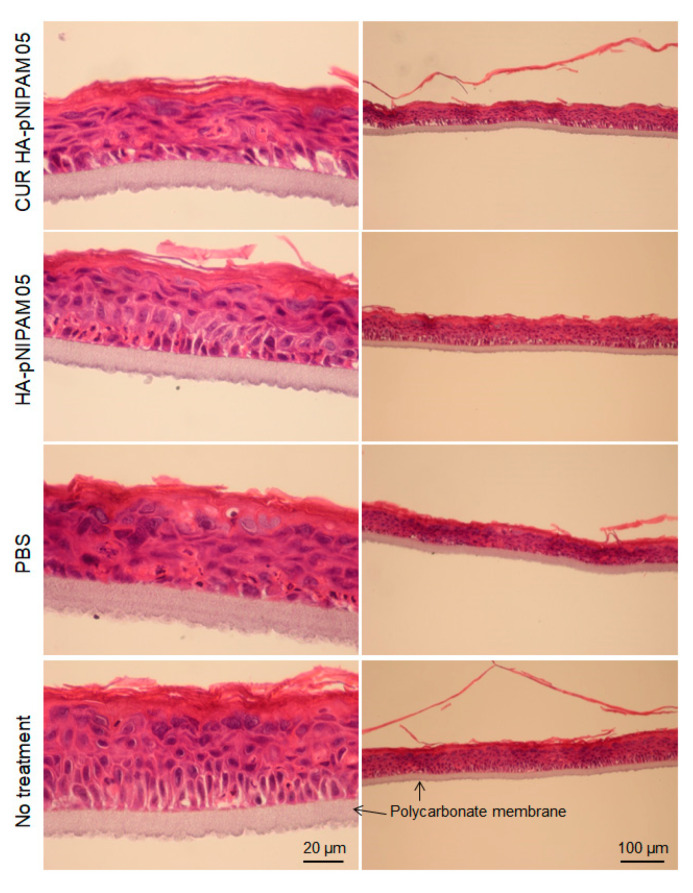
Histological analysis for acute skin irritation test. Hematoxylin and Eosin (H&E) stained EpiSkin^®^. The scale bars for each left and right column indicate 20 µm and 100 µm, respectively.

The skin irritation of the nanogel formulations was also investigated using the SkinEthic™ model. The SkinEthic™ model represented human tissue construct on an inert polycarbonate filter and trans-well plastic ring support in which normal, human-derived keratinocytes were cultured and differentiated to resemble a three-dimensional epidermis with 0.5 cm^2^ surface area [34,35]. Histological observations were performed with the model treatment of CUR-HA-pNIPAM 05 nanogel, HA-pNIPAM 05 nanogel without the drug, nanogel vehicle (PBS) control, and the skin model with no treatment as a negative control. From Figure 5, it can be derived that the tissue integrity between the polycarbonate membrane and stratum basal was compromised in all samples treatment that could be from the handling process, so as the topmost layer of stratum corneum. However, when comparing the overall tissue after treatments, the models treated with nanogel samples loaded with and without curcumin had similar structures and those comparable to the negative control. To our surprise, the comparatively most serious tissue damage was observed with the model treated with PBS, which is considered the polar solvent. Stratum corneum was thinner in the swollen cells and the disrupted tissue structure underneath. The possible explanation for this finding was that the treatments could compromise the contact and the exchange for the skin model to the required nutrients and growth factors from its special and appropriate culture medium. While the PBS treatment created serious damage, the HA-pNIPAM nanogel system could only prevent those affected from the vehicle treatment even when loaded with the drug. This observation could be from the protection and moisturization effect caused by the backbone polymer (HA) used in the nanogel formulations [36].

### 3.5. In Vitro Cytotoxicity of Curcumin in the Nanogel Formulation

**Figure 6:**
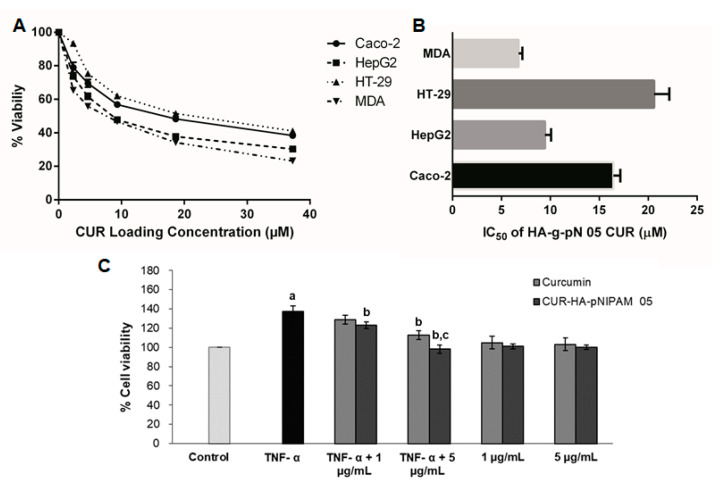
(**A**) The cell viability of four different cancer cell lines per increasing doses of curcumin in HA-pNIPAM 05 nanogel formulation. (**B**) IC_50_ of curcumin encapsulated in the nanogel formulation against four different cancer cell lines; (**C**) the anti-proliferation assay of curcumin and CUR-HA-pNIPAM 05 (1 and 5 µg/mL) against TNF-α-induced HaCaT cell proliferation. Data presented are mean ± SD values of the four replications. ^a^ *p* < 0.05 indicates significant differences from the control group; ^b^ *p* < 0.05 indicates significant differences from the TNF-α group; ^c^ *p* < 0.05 indicates significant differences from the TNF-α + curcumin 5 µg/mL group.

The cytotoxicity assays for the treatment of CUR-HA-pNIPAM 05 on four cancer cell lines, including MDA-MB-231, HT-29, HepG2, and Caco-2 are presented in Figure 6A,B. The graphs represent the viability data resulting from the titration of different dilutions of each CUR-HA-pNIPAM 05 treated on each cell type. The overall relationship between cytotoxicity and drug concentrations across all experimental cell lines was dose-dependent (Figure 6A). The corresponding IC_50_ for each cell line was calculated and shown in Figure 6B. The IC_50_ of curcumin control treatment dissolved in DMSO for MDA-MB-231, HT-29, HepG2, and Caco-2 were 5.29 ± 0.22, 10.20 ± 0.96, 13.41 ± 1.16, and 12.24 ± 1.08, respectively, with replicated analysis (n = 4). The resulted IC_50_ of curcumin in nanogel towards these cell lines were slightly higher than that of curcumin in DMSO except for the HepG2 cell, yet still in a comparably effective range when tested on the same cell lines. However, with CUR-HA-pNIPAM 05 formulation, the aqueous-based formulation could be beneficial considering the physiological compatibility of the product to be administered.

### 3.6. Anti-Psoriatic Effect of the Nanogel Formulation

The TNF-α-induced proliferation of HaCaT cells was used as a model to imitate the hyperproliferation of keratinocytes in psoriasis. The anti-psoriatic effect of the curcumin and curcumin in the nanogel is shown in Figure 6C. The TNF-α-induced group had significantly increased cell proliferation by 40% compared to the control group (*p* < 0.05), indicating that the TNF-α treatment is suitable for a psoriasis model. The treatment of curcumin at 1 µg/mL and 5 µg/mL to the TNF-α-induced cells inhibited the proliferation by 6% and 18%, respectively, while the treatment of CUR-HA-pNIPAM 05 at an equivalent amount of curcumin decreased cell proliferation by 10% and 30%, respectively. This could be attributed to the improved cellular uptake by the nanogel formulation. Therefore, the nanogel formulation fabricated from 0.5% HA-pNIPAM could enhance the anti-proliferative activity of curcumin, which might be used in the topical drug formulation for the treatment of psoriasis and other proliferative skin diseases.

## 4. Conclusions

In this study, a curcumin-loaded thermo-responsive nanogel was developed from pNIPAM-grafted HA to overcome the limitation of its aqueous solubility for therapeutic application. We first studied the effect of pNIPAM grafting on the nanogel assembly of HA. Then, the nanogel formulation of 0.5% pNIPAM-grafted HA polymer was tested for the drug-loading capacity and biocompatibility. Experiments in NIH-3T3 cells showed that the nanogel formulation was cytocompatible, suggesting the safety of this nano-formulation. The nanogel formulation was investigated for skin irritation via histopathological study with an artificial skin equivalence model, and it was found to possess the skin protective property. Additionally, this curcumin-loaded nanogel formulation delivered curcumin into NIH-3T3 cells, as shown by confocal microscopy. Finally, the in vitro preliminary pharmacological activities of curcumin nanogel was evaluated and confirmed for its effectiveness against cancer cells and hyperproliferative keratinocytes. Therefore, this nano delivery system has the potential to improve the bioavailability of low-solubility drugs and can be applied to other pharmaceuticals as a biocompatible aqueous-based delivery system.

## Data Availability

The data presented in this study are available in the supplementary material.

## Supplementary Materials

The following are available online at https://www.mdpi.com/2073-4360/13/2/194/s1. Figure S1. ^1^H NMR spectra of pNIPAM grafted hyaluronic acid with 5% degree of modification by a molecule of pNIPAM per total reacting HA monomers. The degree of modification of grafted HA was calculated from the ratio integrations of the peak marked with the asterisk representing the two protons on C1 of pNIPAM backbone, and the subtraction of the combined integration of the peak represent protons on *N*-acetyl group of the glucosamine ring with the single proton attached to the chiral backbone of pNIPAM in 1:2 proportional to the previous peak represent protons on another backbone’s carbon designated with an arrow. The top and the bottom panels showed different reaction times for grafting in which the degree of grafting was achieved; Figure S2. CellTiter Blue cell viability assay shows the relative non-toxicity of all nanogel formulations based on 0.5% *w*/*v* polymer solutions of HA-pNIPAM with and without curcumin tested on NIH-3T3 cells at 48 h treatment. No significant changes in cell viability were observed across most treatment dilutions (*p* < 0.05) except for the 10 times dilution. The values expressed are means ± SD, n = 3.
