# Supplementary material for: Self-Assembled Thermoresponsive Nanogel from Grafted Hyaluronic Acid as a Biocompatible Delivery Platform for Curcumin with Enhanced Drug Loading and Biological Activities
Source: Polymers (Basel). 2021 Jan 7;13(2):194. doi: 10.3390/polym13020194 (PMC7825653; doi:10.3390/polym13020194)
Supplement: Supplementary file 1 [file polymers-13-00194-s001.pdf]

polymers

MDPI

Supplementary File

Self-Assembled Thermoresponsive Nanogel from Grafted Hyaluronic Acid as a Biocompatible Delivery Platform for Curcumin with Enhanced Drug Loading and Biological Activities

Jittima Amie Luckanagul, Pahweenvaj Ratnatilaka Na Bhuket, Chawanphat Muangnoi, Pranee Rojsitthisak, Qian Wang, Pornchai Rojsitthisak

Supplementary Information

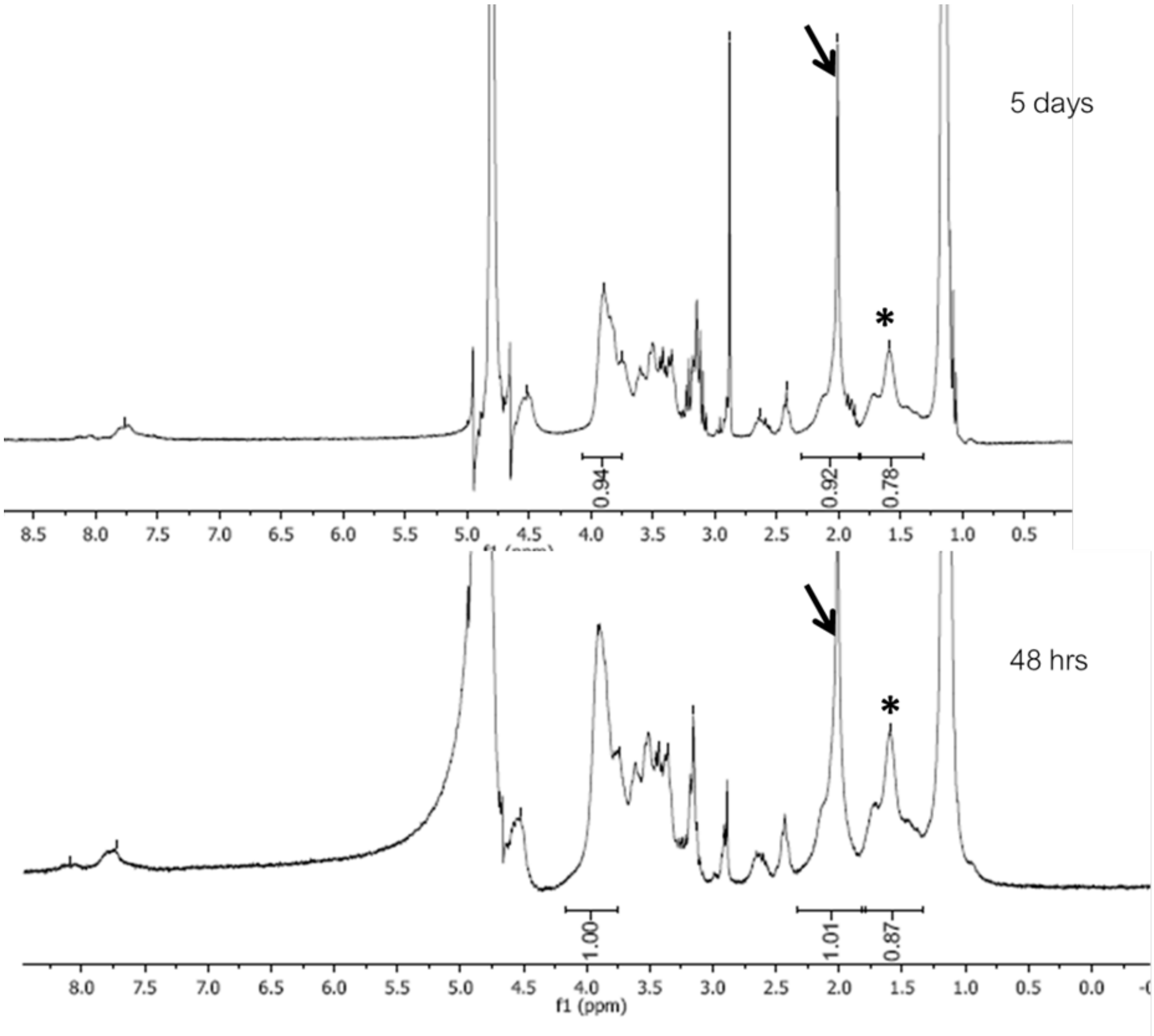

Figure S1. ^{1}H NMR spectra of HA-pNIPAM with 5% degree of modification by the molecule of pNIPAM per total reacting HA monomers. The degree of modification of g HA-pNIPAM grafted HA was calculated from the ratio integrations of the peak marked with the asterisk representing the 2 protons on C1 of pNIPAM backbone

and the subtraction of the combined integration of the peak represent protons on *N*-acetyl group of the glucosamine ring with the single proton attached to the chiral backbone of pNIPAM in 1:2 proportional to the previous peak represent protons on another backbone's carbon designated with the arrow. The top and the bottom panels showed different reaction times for grafting in which the degree of grafting was achieved.

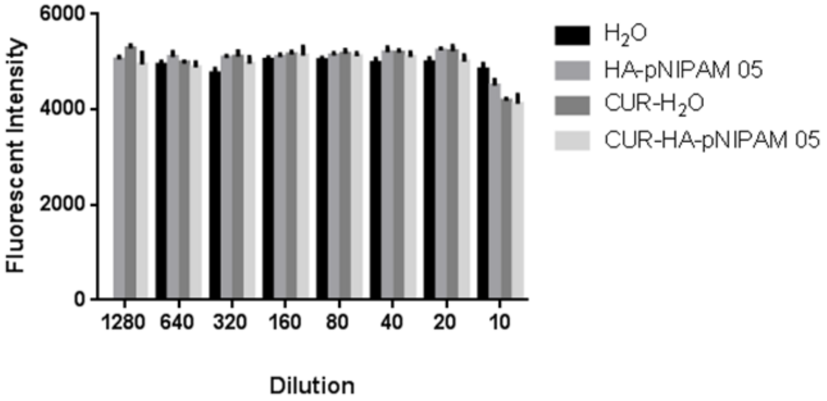

Figure S2. CellTiter Blue cell viability assay showed the relative non-toxicity of all nanogel formulations based on 0.5% w/v polymer solutions of HA-pNIPAM with and without curcumin tested on NIH-3T3 cells at 48 h treatment. No significant changes in cell viability were observed across most treatment dilutions ($p < 0.05$) except for the 10 times dilution. The values expressed are means ± SD, n = 3.
